# Polycystic ovary syndrome and risk of adverse obstetric outcomes: a retrospective population-based matched cohort study in England

**DOI:** 10.1186/s12916-022-02473-3

**Published:** 2022-08-30

**Authors:** Anuradhaa Subramanian, Siang Ing Lee, Katherine Phillips, Konstantinos A. Toulis, Punith Kempegowda, Michael W. O’Reilly, Nicola J. Adderley, Shakila Thangaratinam, Wiebke Arlt, Krishnarajah Nirantharakumar

**Affiliations:** 1 Institute of Applied Health Research, University of Birmingham, Birmingham, UK; 2 Institute of Metabolism and Systems Research, WHO Collaborating Centre for Global Women’s Health, University of Birmingham, Birmingham, UK; 3 Endocrinology and Metabolism Unit, Department of Medicine, Royal College of Surgeons in Ireland (RCSI), University of Medicine and Health Sciences, Dublin, Republic of Ireland; 4 Birmingham Women’s and Children’s NHS Foundation Trust, Birmingham, UK; 5 National Institute for Health Research (NIHR) Birmingham Biomedical Research Centre, University Hospitals Birmingham NHS Foundation Trust and University of Birmingham, Birmingham, UK; 6 Midlands Health Data Research UK, Birmingham, UK

**Keywords:** Polycystic ovary syndrome, PCOS, Preterm, Birthweight, Stillbirth, Delivery

## Abstract

**Background:**

Polycystic ovary syndrome (PCOS) affects up to one in five women of childbearing age. Observational studies assessing the association between maternal PCOS and adverse obstetric outcomes have reported varying results, depending on patient population, diagnostic criteria for PCOS and covariates accounted for in their analyses. We aimed to assess the risk of obstetric outcomes among a population-based representative cohort of women with PCOS compared to an age-matched cohort of women without PCOS.

**Methods:**

A retrospective cohort study was conducted of pregnancies of women in England aged 15–49 years identified from the Clinical Practice Research Datalink (CPRD) GOLD pregnancy register and linked Hospital Episodes Statistic (HES) data between March 1997 and March 2020. Pregnancies from the register that had a linked HES delivery record were included. Linked CPRD primary care data was used to ascertain maternal PCOS exposure prior to pregnancy. To improve detection of PCOS, in addition to PCOS diagnostic codes, codes for (1) polycystic ovaries or (2) hyperandrogenism and anovulation together were also considered. Sensitivity analysis was limited to only pregnant women with a diagnostic code for PCOS.

Primary outcomes ascertained from linked HES data were (1) preterm delivery (gestation < 37 weeks), (2) mode of delivery, (3) high (> 4000 g) or low birthweight (< 2500 g) and (4) stillbirth. Secondary outcomes were (1) very preterm delivery (< 32 weeks), (2) extremely preterm delivery (< 28 weeks), (3) small and (4) large for gestational age.

Conditional logistic regression models were performed adjusting for age, ethnicity, deprivation, dysglycaemia, hypertension, thyroid disorders, number of babies born at index pregnancy, and pre-gravid BMI. Multiple imputation was performed for missing outcome data.

**Results:**

27,586 deliveries with maternal PCOS were matched for age (± 1 year) to 110,344 deliveries without PCOS. In the fully adjusted models, maternal PCOS was associated with an increased risk of (1) preterm birth [aOR: 1.11 (95% CI 1.06–1.17)], and (2) emergency caesarean, elective caesarean and instrumental vaginal compared to spontaneous delivery [aOR: 1.10 (1.05–1.15), 1.07 (1.03–1.12) and 1.04 (1.00–1.09), respectively]. There was absence of association with low birthweight, high birthweight and stillbirth. In the sensitivity analysis, the association with preterm birth [aOR: 1.31 (95% CI 1.13–1.52)], emergency caesarean [aOR: 1.15 (95% CI 1.02–1.30)], and elective caesarean [aOR: 1.03 (95% CI 1.02–1.03)] remained.

While there was no significant association with any of the secondary outcomes in the primary analysis, in the sensitivity analysis maternal PCOS was associated with increased risk of extremely preterm delivery [aOR: 1.86 (95% CI 1.31–2.65)], and lower risk of small for gestational age babies [aOR: 0.74 (95% CI 0.59–0.94)].

**Conclusions:**

Maternal PCOS was associated with increased risk of preterm and caesarean delivery. Association with low birthweight may be largely mediated by lower gestational age at birth.

**Supplementary Information:**

The online version contains supplementary material available at 10.1186/s12916-022-02473-3.

## Background

Polycystic ovary syndrome (PCOS) is a common yet underdiagnosed endocrine disorder [1, 2], with a diagnosed prevalence of 10% [3], it is estimated that half of women with PCOS are undiagnosed [4]. Consensus criteria for diagnosis of PCOS require presence of two out of the following three features: (i) biochemical evidence or clinical manifestations of androgen excess such as hirsutism and hair loss, (ii) chronic oligo-/anovulation and (iii) polycystic ovarian morphology on ultrasound [5]. The adverse clinical phenotype is largely driven by a complex interplay between insulin resistance and androgen excess [6]. PCOS is considered a lifelong metabolic disorder [7] with a plethora of adverse risks during and following pregnancy [8], and even posing intergenerational risks to the children of women with PCOS [9]. These risks may be attributed to the biochemical features of PCOS or several other co-existing risk factors such as high BMI, or comorbidities that are commonly seen among women with PCOS [10].

Several systematic reviews have pooled together findings from observational studies examining the association between maternal PCOS and the risk of a range of obstetric outcomes. However, these reviews suggest varying results across the primary studies that they included owing to methodological heterogeneity [11–13], which included differences in terms of source population, criteria employed for PCOS ascertainment, and confounders matched and adjusted for in their design and analysis respectively. Several of these primary studies are further limited in terms of outdated data, their sample size [14, 15], and restrictive selection of pregnant women who have undergone assisted reproduction [16, 17] within their studies.

Furthermore, socio-demographic factors such as high BMI, deprivation and minority ethnic background, as well as metabolic disturbances such as insulin resistance, hypertension and thyroid disorders, may exacerbate the severity of PCOS [2, 18–22]. The existing literature is limited in terms of comprehensively identifying, assessing and accounting for these confounders/mediators.

Therefore, in order to overcome the limitations of the observational studies in the existing literature, we have performed an age-matched retrospective cohort study of pregnant women using a population representative, UK primary care-based data source, to identify the risk of adverse obstetric outcomes including preterm birth, a different mode of delivery, high and low birthweight, and stillbirth in women with PCOS compared to those without. Furthermore, we adjusted for confounders agreed a priori, in a series of regression models adding covariates step by step to identify the extent of confounding conferred by each risk factor.

## Methods

### Study design and data source

A retrospective open cohort study of pregnant women identified from primary care records [Clinical Practice Research Datalink (CPRD) GOLD Pregnancy Register], with their delivery recorded in secondary care [linked Hospital Episode Statistics (HES)] between 1997 and 2020, was performed to determine the incidence of adverse obstetric outcomes among women with PCOS in comparison to women without PCOS.

CPRD GOLD contains representative data from 7% of the general practices across the UK, covering 20 million patients from 973 practices. It contains pseudo-anonymized patient-level data on demographics, symptoms, diagnoses, drug prescriptions, physical measurements, and laboratory test results. Furthermore, patient-level data can be linked to other data sources such as HES data and deprivation data, via a trusted third party [23]. The linkage of databases aided capture of information on exposure (PCOS) from primary care, the obstetric outcomes from HES maternity tail and important potential confounders from both primary and secondary care. Symptoms and diagnoses are recorded within CPRD GOLD using Read codes, a hierarchical clinical coding system. Using maternity, antenatal and delivery health records within CPRD GOLD, pregnancy episodes and their outcomes are identified through a validated algorithm [24], which formulated the CPRD GOLD Pregnancy Register and formed the source cohort for our study.

### Study population

Pregnant women were included from the CPRD GOLD Pregnancy Register if they were registered at a general practice in England and had a record of delivery from linked HES data (containing information on admissions to National Health Service (NHS) hospitals in England).

Deliveries formed the unit of analysis in our study and an index date was assigned to each eligible delivery record. Women with implausible data linkage (where a patient record in HES is linked to more than 20 patient records across 20 different primary care practices) were excluded. Furthermore, delivery records were excluded if they were (1) duplicates or (2) misclassified miscarriage, postnatal or antenatal record. Delivery records were considered misclassified miscarriages if the reported gestational age was less than 23 weeks. If two deliveries were recorded within 180 days of each other for the same patient, one of the delivery records was considered as a misclassified antenatal or postnatal record. Finally, delivery records were excluded if women were ineligible or were lost to follow-up within primary care at the delivery. Patients were considered ineligible within primary care if they (1) did not have an acceptable patient flag within CPRD GOLD (indicating sufficient data quality), (2) did not have a minimum registration period of 1 year with an eligible general practice on delivery date (practices were considered eligible one year after the “up-to-standard” date, a flag for sufficient practice data quality) and (3) were aged < 15 or > 49 years on delivery date.

Once linked, the mother’s PCOS exposure status for each delivery record was ascertained from primary care prior to the index date (date of delivery). PCOS was defined as a Read code record of PCOS. Due to underdiagnosis of PCOS within primary care, we also considered records of polycystic ovaries (PCOs) [20, 25], or a combination of symptom codes indicating a missed PCOS diagnoses based on Rotterdam criteria [(1) anovulation and (2) biochemical or symptomatic presentation of hyperandrogenism; a Read code record of hair loss or hirsutism and a recorded measure of serum testosterone level ≥ 2.0 nmol/L were considered as symptomatic and biochemical presentation of hyperandrogenism, respectively].

**Fig. 1:**
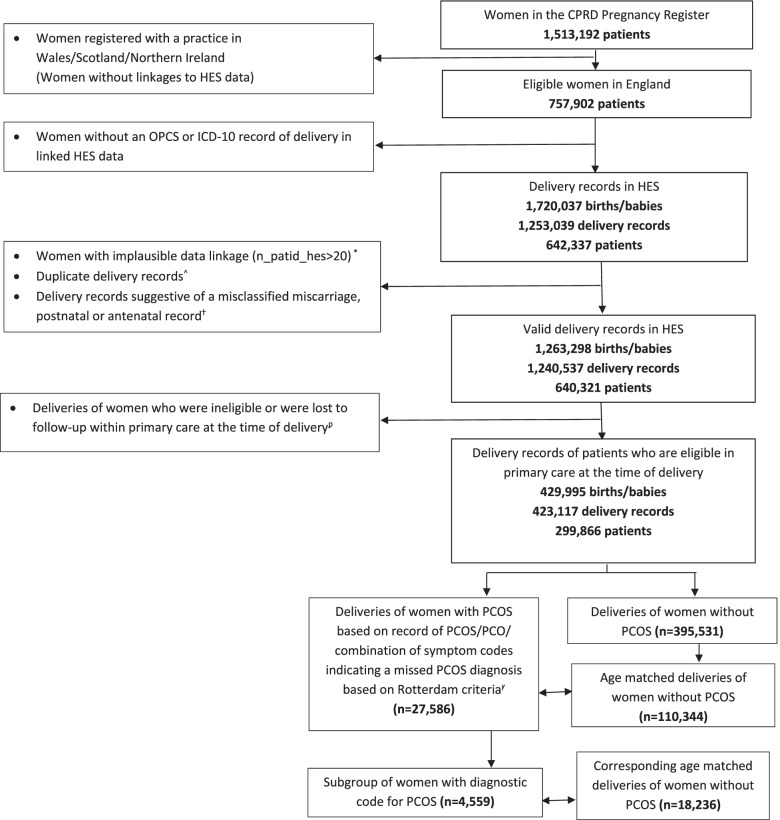
Flow chart describing cohort selection * Number of primary care patient records linked to the same HES patient record is large (n_patid_hes>20). This linkage may not be reliable and therefore these patients are excluded ^ (1) In case of more than 9 births during the same delivery with missing birthweight data, only the first birth is included and the rest are considered duplicates; (2) In case of multiple births, if all babies have the same birthweight recorded, then only one of the babies is included and the rest are considered duplicates; (3) If the number of births reported within a delivery does not match with the number of birth records within a delivery, excess birth records are considered duplicates. Duplicates are excluded U+2D15 Delivery records are considered as misclassified miscarriages if the reported gestational age is less than 23 weeks; Delivery records are considered as misclassified antenatal or postnatal records if two deliveries are recorded within 180 days of each other for the same patient, and the record with missing birthweight is considered misclassified (1) Patients without an acceptable patient flag within CPRD GOLD (indicating sufficient data quality); (2) Patients without a minimum registration period of one year with an eligible general practice on delivery date (Practices were considered eligible one year after the “up-tostandard” date, a flag for sufficient practice data quality); (3) Patients aged <15- or >49 on delivery date; (4) Patients transferred out of practice, or their registered practice stopped contributing data to CPRD GOLD on their date of delivery Rotterdam criteria: (1) anovulation and (2) biochemical or symptomatic presentation of hyperandrogenism; Read code record of hair loss or hirsutism and a recorded measure of serum testosterone level ≥ 2.0 nmol/L was considered as symptomatic and biochemical presentation of hyperandrogenism respectively

For each delivery record of women with PCOS (in a random order), we randomly selected four control delivery records of women without PCOS from a pool of age-matched (± 1 year) pregnant women without replacement. Cohort selection for this study is described in Fig. 1.

### Outcomes

We considered four primary outcomes identified from HES data: (1) preterm birth, (2) mode of delivery, (3) high or low birthweight and (4) stillbirth.

Gestational age recorded within the HES maternity tail at the time of delivery and relevant ICD-10 codes were used to identify the outcome preterm birth (gestational age at birth < 37 weeks). Based on Operating Procedure Codes Supplement (OPCS) codes and ICD-10 codes, we classified mode of delivery into one of the following four categories as a categorical outcome variable: (1) emergency caesarean section, (2) elective or other unspecified caesarean section, (3) instrumental vaginal delivery and (4) spontaneous or other unspecified vaginal delivery (reference category). Based on birthweight(s) recorded in the maternity tail, we classified the delivery as high or low birthweight delivery if at least one of the babies born in that delivery was above 4000 g or below 2500 g, respectively. In addition, a record of the relevant ICD-10 code was used to identify a high birthweight baby. Stillbirth outcomes were identified using relevant ICD-10 codes and from maternity tail records.

As secondary outcomes, we further classified gestational age to identify very preterm (< 32 weeks) and extremely preterm (< 28 weeks) delivery. Small and large for gestational age babies (birthweight < 10th and > 90th centile, respectively) were identified using the INTERGROWTH 21st project [26], and their software tools, by comparing the birthweight and gestational age recorded in HES data to the international anthropometric standards.

### Explanatory variables

We considered risk factors or features of PCOS that are also obstetric risk factors as possible explanatory variables and adjusted for them in our analysis in a step-by-step manner. This included age, ethnicity, deprivation, impaired glucose regulation based on a diagnosis of type 2 diabetes or prediabetes, diagnosis of hypertension, thyroid disorders, number of babies born within the delivery, and pre-gravid body mass index (BMI). For the outcomes low and high birthweight and mode of delivery, we further considered gestational age as an explanatory variable.

Ethnicity was identified using relevant Read codes from primary care records and was categorized as (1) white Caucasian, (2) South Asian, (3) black Afro-Caribbean and (4) mixed or multiple ethnic group or (5) other ethnic minority groups. Primary care linked English index of multiple deprivation (IMD) data provided a relative measure of deprivation based on seven different domains [27]. Type 2 diabetes was identified from primary care through relevant Read Codes, record of HbA1c ≥ 48 mmol/L (≥ 6.5%) or fasting blood glucose > 7 mmol/L. Impaired glucose regulation was identified through relevant Read codes, HbA1c ≥ 42 mmol/L (≥ 6.0%) or fasting blood glucose ≥ 5.5 mmol/L. Diagnoses of hypertension and thyroid disorders were identified from primary care through Read code records. The number of babies born during that delivery was derived from linked HES maternity tail records. Pre-gravid BMI was identified as the latest BMI measured in primary care at least a year before index date and was categorized according to WHO standards as under/normal weight (< 25 kg/m^2^), overweight (25–30 kg/m^2^) and obese (≥ 30 kg/m^2^). A separate missing category was created for those with missing data on ethnicity, deprivation, number of babes born within the delivery and pre-gravid BMI.

### Statistical analysis

Deliveries were the unit of our analysis. Baseline explanatory variables were described using appropriate summary statistics stratified by exposure to maternal PCOS. Mean with standard deviation (SD) and median with interquartile range (IQR) were provided for continuous variables as appropriate. Frequency and percentage were provided for categorical variables.

Multiple imputation using chained equation was performed to impute missing delivery related data that were essential to compute outcome variables [28–30]. Missing values were imputed 31 times (since gestational age was missing among 31% of the women in the study) using linear (for gestational age and birthweight outcomes), logistic (for stillbirth outcome and sex of the baby) and multinomial logistic (for delivery method categorical outcome) regression as appropriate using the variables age, BMI, impaired glucose regulation, deprivation and the number of babies delivered. Conditional logistic or multinomial logistic regression models were used to provide unadjusted and adjusted odds ratios (ORs) for the binary and nominal categorical outcome variables (mode of delivery), respectively, among women with PCOS compared to women without PCOS. We estimated robust confidence intervals after accounting for the intragroup correlation of multiple deliveries of a woman throughout her reproductive age. We included the explanatory variables in a step-by-step manner in the regression model, resulting in a fully adjusted model.

A sensitivity analysis was performed restricting to women with a coded diagnosis of PCOS only and their corresponding matched controls. All analyses were performed in Stata IC version 15. Two-sided P values were obtained for all tests, and a *P* value < 0.05 was considered as statistically significant. Selection of Read, ICD-10 and OPCS code lists was performed using an inhouse developed software platform called Code Builder, with systematic searching of existing code lists, and through clinical knowledge and discussion methods used in our previous publications [31], and the list of codes used for exposure and outcome ascertainment are provided in Additional files 1 and 2. The study results are reported as per the RECORD (REporting of studies Conducted using Observational Routinely-collected health Data) statement.

## Results

Out of the 1,513,192 women identified within the CPRD GOLD Pregnancy Register, 757,902 women were eligible for linkage to HES. Of these women, 642,337 had a record of delivery (*n* = 1,253,039) within HES linked data based on OPCS and ICD-10 records. After excluding patients and delivery records as outlined in the “Methods” section above (Fig. 1), a final eligible cohort of 423,117 delivery records from 299,866 patients was identified.

From the eligible cohort of delivery records, 27,586 (6.5%) were for women with a coded diagnosis of PCOS/PCO or a combination of symptom codes indicating a missed PCOS diagnosis based on Rotterdam criteria; these deliveries formed the exposed cohort for our primary analysis. From a pool of 395,531 control delivery records, an unexposed cohort of 110,344 was selected, matched for maternal age. In the sensitivity analysis, 4559 (1.1%) deliveries by women who had a specifically coded diagnosis for PCOS, and their corresponding matched controls (18,236 deliveries) were included.

### Baseline characteristics

The mean (SD) age at delivery of women with and without PCOS was 30.86 (5.38) and 30.85 (5.33), respectively (Table 1).

**Table 1:** Baseline characteristics of women with PCOS and age-matched controls

	**Primary analysis**	
Variables	Deliveries of women with PCOS*	**Age-matched deliveries of women without PCOS**
**All**	**(*n* = 27,586)**	**(*n* = 110,344)**
**Age at delivery [mean (SD)]**	30.86 (5.38)	30.85 (5.33)
**Age at delivery [median (IQR)]**	30.00 (26.00–34.00)	31.00 (27.00–34.00)
**Age categories, ***n***(%)**		
** 14–19 years**	467 (1.69)	1802 (1.63)
** 20–29 years**	11,537 (41.82)	45,596 (41.32)
** 30–39 years**	14,357 (52.04)	58,313 (52.85)
** 40–50 years**	1225 (4.44)	4633 (4.20)
**Pre-gravid BMI [mean (SD)]**	26.54 (6.38)	25.11 (5.43)
**Pre-gravid BMI [median (IQR)]**	24.00 (21.00–30.00)	23.00 (21.00–27.00)
**BMI categories, ***n***(%)**		
** < 25 kg/m**^2^	13,055 (47.32)	59,799 (54.19)
** 25–29.9 kg/m**^2^	6493 (23.54)	25,103 (22.75)
** 30–34.9 kg/m**^2^	3667 (13.29)	10,423 (9.45)
** 35–39.9 kg/m**^2^	1882 (6.82)	4023 (3.65)
** ≥ 40 kg/m**^2^	1013 (3.67)	2002 (1.81)
** Missing**	1476 (5.35)	8994 (8.15)
**IMD, ***n***(%)**		
** 1 (most deprived)**	3334 (12.09)	12,989 (11.77)
** 2**	2795 (10.13)	11,052 (10.02)
** 3**	2706 (9.81)	11,215 (10.16)
** 4**	2637 (9.56)	11,031 (10.00)
** 5**	2973 (10.78)	11,782 (10.68)
** 6**	2578 (9.35)	10,165 (9.21)
** 7**	2547 (9.23)	10,557 (9.57)
** 8**	2696 (9.77)	10,319 (9.35)
** 9**	2693 (9.76)	10,353 (9.38)
** 10 (least deprived)**	2607 (9.45)	10,793 (9.78)
** Missing**	19 (0.07)	81 (0.07)
**Ethnicity, ***n***(%)**		
** White**	13,343 (48.37)	50,894 (46.12)
** South Asian**	1465 (5.31)	3638 (3.30)
** Black Afro-Caribbean**	1567 (5.68)	5315 (4.82)
** Mixed or multiple ethnicity**	170 (0.62)	651 (0.59)
** Others**	721 (2.61)	2561 (2.32)
** Missing**	10,320 (37.41)	47,285 (42.85)
**Record of symptoms and measurements at baseline, ***n***(%)**		
** PCO**	12,706 (46.06)	0 (0)
** Hair loss**	2898 (10.51)	2645 (2.40)
** Hirsutism**	1825 (6.62)	645 (0.58)
** Anovulation**	17,852 (64.71)	10,845 (9.83)
** High testosterone (serum testosterone level ≥ 2.0 nmol/L)**	3250 (11.78)	467 (0.42)
**Other comorbidities, ***n***(%)**		
** Type 2 diabetes**	675 (2.45)	1259 (1.14)
** Prediabetes**	1123 (4.07)	2038 (1.85)
** Hypertension**	489 (1.77)	1230 (1.11)
** Thyroid disorders**	1105 (4.01)	2403 (2.18)
**Number of babies at the delivery,***n***(%)**		
** 1**	27,163 (98.47)	108,446 (98.28)
** 2**	409 (1.48)	1864 (1.69)
** 3**	14 (0.05)	30 (0.03)
** 4**	0 (0)	4 (0.00)

^*^Record of PCOS/PCO/conglomeration of symptom codes indicating a missed PCOS diagnosis based on Rotterdam criteria [two of the three symptoms recorded: (1) PCO, (2) anovulation and (3) biochemical or symptomatic presentation of hyperandrogenism]. Read code record of hair loss or hirsutism and a recorded measure of serum testosterone level ≥ 2.0 nmol/L was considered as symptomatic and biochemical presentation of hyperandrogenism, respectively]

*PCOS* Polycystic ovary syndrome, *PCO* Polycystic ovaries, *SD* Standard deviation, *IQR* Interquartile range, *BMI* Body Mass Index, *IMD* Index of multiple deprivation

Compared to women without PCOS, women with PCOS had higher pre-gravid BMI [mean (SD): 26.54 (6.38) vs 25.11 (5.43)], were more likely to be deprived (IMD most deprived decile (1): 12.09% vs 11.77%) and were more likely to be from an ethnic minority [South Asian (5.31% vs 3.30%) and black Afro-Caribbean (5.68% vs 4.82%)]. As expected, women with PCOS were more likely to have a record of PCOS-related symptoms such as hair loss (10.51% vs 2.40%), hirsutism (6.62% vs 0.58%), anovulation (64.71% vs 9.83%), and serum testosterone ≥ 2.0 nmol/L (11.78% vs 0.42%). Women with PCOS were also more likely to have metabolic disturbances including comorbidities such as type 2 diabetes (2.45% vs 1.14%), prediabetes (4.07% vs 1.85%), hypertension (1.77% vs 1.11%), and thyroid disorders (4.01% vs 2.18%) (Table 1). The baseline characteristics of deliveries of women with a diagnostic code for PCOS and their maternal age-matched deliveries of women without PCOS are presented in Additional file 3.

### Risk of primary obstetric outcomes among women with PCOS compared to their age-matched controls

#### Preterm birth

Among the delivery records of women with and without a pre-existing diagnosis of PCOS, 7.63% (*n* = 2104) and 6.82% (*n* = 7520) of them were delivered preterm, resulting in 13% increased crude odds of preterm delivery among women with PCOS compared to women without PCOS [OR 1.13 (95% CI 1.07–1.19)] (Table 2). There was marginal attenuation of the increased odds with adjustment for covariates [aOR: 1.11 (1.06–1.17)]. For the secondary outcomes of preterm delivery, among the delivery records of women with and without PCOS, 2.24% and 2.03% of deliveries were before 32 weeks of gestational age and 0.99% and 0.82% were before 28 weeks of gestational age, respectively (Table 3). There were 11% and 20% increased crude odds of delivery before 32 and 28 weeks of gestational age, respectively [OR 1.11 (95% CI 1.01–1.22) and 1.20 (95% CI 1.04–1.39)], among women with PCOS compared to women without PCOS. There was marginal attenuation in the effect size at each step when serially adjusting for covariates, which resulted in increased odds of both outcomes among women with PCOS compared to women without PCOS, although statistically insignificant in the final model [aOR: 1.07 (0.97–1.18) and 1.13 (0.98–1.29) for delivery < 32 and < 28 weeks of gestational age, respectively]. In the sensitivity analysis including a sub-cohort of deliveries by women with a diagnostic code for PCOS and their corresponding maternal age-matched control deliveries, the odds ratios were more pronounced for delivery less than 37, 32 and 28 weeks of gestational age [gestational age < 37 weeks aOR: 1.31 (1.13–1.52); gestational age < 32 weeks aOR: 1.42 (0.88–2.31); gestational age < 28 weeks aOR: 1.86 (1.31–2.65)] (Additional files 4 and 5).

**Table 2:** Risk of primary obstetric outcomes among women with PCOS compared to women without PCOS

Outcomes	Deliveries of women with PCOS* (*n* = 27,586)	Age-matched deliveries of women without PCOS (*n* = 110,344)
**Preterm (< 37 weeks of gestational age at delivery)**		
** Number of patients**	27,586	110,344
** Outcome events, ***n***(%)**	2104 (7.63%)	7520 (6.82%)
** Unadjusted OR (95% CI)**	1.13 (1.07–1.19)	
** Adjusted OR (95% CI) (Model 1)**	1.12 (1.07–1.17)	
** Adjusted OR (95% CI) (Model 2)**	1.09 (1.03–1.14)	
** Adjusted OR (95% CI) (Model 3)**	1.11 (1.05–1.17)	
** Adjusted OR (95% CI) (Model 4)**	1.11 (1.06–1.17)	
**Mode of delivery**		
** Number of patients**	27,586	110,344
**Outcome events, ***n***(%)**		
** Emergency CS**	3473 (12.59%)	12,073 (10.94%)
** Elective/other/unspecified CS**	4211 (15.26%)	15,279 (13.85%)
** Instrumental vaginal**	3077 (11.15%)	12,573 (11.39%)
** Spontaneous/other/unspecified vaginal**	16,825 (60.99%)	70,419 (63.82%)
**Unadjusted OR (95% CI)**		
** Emergency CS**	1.20 (1.15–1.26)	
** Elective/other/unspecified CS**	1.15 (1.11–1.20)	
** Instrumental vaginal**	1.02 (0.98–1.07)	
** Spontaneous/other/unspecified vaginal**	Ref	
**Adjusted OR (95% CI) (Model 1)**		
** Emergency CS**	1.20 (1.15–1.26)	
** Elective/other/unspecified CS**	1.15 (1.10–1.19)	
** Instrumental vaginal**	1.02 (0.98–1.07)	
** Spontaneous/other/unspecified vaginal**	Ref	
**Adjusted OR (95% CI) (Model 2)**		
** Emergency CS**	1.17 (1.12–1.23)	
** Elective/other/unspecified CS**	1.13 (1.08–1.18)	
** Instrumental vaginal**	1.02 (0.98–1.07)	
** Spontaneous/Other/Unspecified Vaginal**	Ref	
**Adjusted OR (95% CI) (Model 3)**		
** Emergency CS**	1.18 (1.12–1.23)	
** Elective/other/unspecified CS**	1.13 (1.09–1.18)	
** Instrumental vaginal**	1.02 (0.98–1.07)	
** Spontaneous/other/unspecified vaginal**	Ref	
**Adjusted OR (95% CI) (Model 4)**		
** Emergency CS**	1.11 (1.06–1.16)	
** Elective/other/unspecified CS**	1.08 (1.04–1.13)	
** Instrumental vaginal**	1.04 (0.99–1.09)	
** Spontaneous/other/unspecified vaginal**	Ref	
**Adjusted OR (95% CI) (Model 5)**		
** Emergency CS**	1.10 (1.05–1.15)	
** Elective/other/unspecified CS**	1.07 (1.03–1.12)	
** Instrumental vaginal**	1.04 (1.00–1.09)	
** Spontaneous/other/unspecified vaginal**	Ref	
**High birthweight > 4 kg (for at least one of the babies)**		
** Number of patients**	27,586	110,344
** Outcome events, ***n***(%)**	2709 (9.82%)	10,632 (9.64%)
** Unadjusted OR (95% CI)**	1.02 (0.97–1.07)	
** Adjusted OR (95% CI) (Model 1)**	1.03 (0.99–1.08)	
** Adjusted OR (95% CI) (Model 2)**	1.03 (0.98–1.07)	
** Adjusted OR (95% CI) (Model 3)**	1.02 (0.98–1.07)	
** Adjusted OR (95% CI) (Model 4)**	0.96 (0.92–1.00)	
** Adjusted OR (95% CI) (Model 5)**	0.97 (0.92–1.01)	
**Low birthweight < 2.5 kg (for at least one of the babies)**		
** Number of patients**	27,586	110,344
** Outcome events, ***n***(%)**	1627 (5.90%)	5903 (5.35%)
** Unadjusted OR (95% CI)**	1.11 (1.05–1.18)	
** Adjusted OR (95% CI) (Model 1)**	1.10 (1.04–1.16)	
** Adjusted OR (95% CI) (Model 2)**	1.08 (1.02–1.14)	
** Adjusted OR (95% CI) (Model 3)**	1.10 (1.03–1.17)	
** Adjusted OR (95% CI) (Model 4)**	1.13 (1.06–1.20)	
** Adjusted OR (95% CI) (Model 5)**	1.03 (0.95–1.13)	
**Stillbirth**		
** Number of patients**	27,586	110,344
** Outcome events, ***n* **(%)**	122 (0.44%)	471 (0.43%)
** Unadjusted OR (95% CI)**	1.04 (0.85–1.26)	
** Adjusted OR (95% CI) (Model 1)**	1.03 (0.85–1.25)	
** Adjusted OR (95% CI) (Model 2)**	1.02 (0.85–1.24)	
** Adjusted OR (95% CI) (Model 3)**	1.01 (0.84–1.22)	
** Adjusted OR (95% CI) (Model 4)**	0.99 (0.81–1.21)	

^*^Record of PCOS/PCO/combination of symptom codes indicating a missed PCOS diagnosis based on Rotterdam criteria [(1) anovulation and (2) biochemical or symptomatic presentation of hyperandrogenism; Read code record of hair loss or hirsutism and a recorded measure of serum testosterone level ≥ 2.0 nmol/L was considered as symptomatic and biochemical presentation of hyperandrogenism, respectively]

*PCOS* Polycystic ovary syndrome, *CS* Caesarean section, *OR* Odds ratio

Model 1: Adjusted for age, ethnicity, and deprivation

Model 2: Adjusted for age, ethnicity, deprivation, baseline dysglycaemia, hypertension and thyroid disorders

Model 3: Adjusted for age, ethnicity, deprivation, baseline dysglycaemia, hypertension, thyroid disorders, and numbers of babies born at the delivery

Model 4: Adjusted for age, ethnicity, deprivation, baseline dysglycaemia, hypertension, thyroid disorders, numbers of babies born at the delivery, and pre-gravid body mass index

Model 5: Adjusted for age, ethnicity, deprivation, baseline dysglycaemia, hypertension, thyroid disorders, numbers of babies born at the delivery, pre-gravid body mass index, and gestational age

**Table 3:** Risk of secondary obstetric outcomes among women with PCOS compared to women without PCOS

Outcomes	Deliveries of women with PCOS*	Age-matched deliveries of women without PCOS
**Very preterm (< 32 weeks of gestational age at delivery)**		
** Number of patients**	27,586	110,344
** Outcome events, ***n***(%)**	619 (2.24%)	2244 (2.03%)
** Unadjusted OR (95% CI)**	1.11 (1.01–1.22)	
** Adjusted OR (95% CI) (Model 1)**	1.09 (0.99–1.19)	
** Adjusted OR (95% CI) (Model 2)**	1.07 (0.97–1.18)	
** Adjusted OR (95% CI) (Model 3)**	1.07 (0.97–1.18)	
** Adjusted OR (95% CI) (Model 4)**	1.07 (0.97–1.18)	
**Extremely preterm (< 28 weeks of gestational age at delivery)**		
** Number of patients**	27,586	110,344
** Outcome events, ***n* **(%)**	272 (0.99%)	909 (0.82%)
** Unadjusted OR (95% CI)**	1.20 (1.04–1.39)	
** Adjusted OR (95% CI) (Model 1)**	1.16 (1.01–1.33)	
** Adjusted OR (95% CI) (Model 2)**	1.14 (0.99–1.31)	
** Adjusted OR (95% CI) (Model 3)**	1.13 (0.98–1.29)	
** Adjusted OR (95% CI) (Model 4)**	1.13 (0.98–1.29)	
**Large for gestational age > 90th percentile (for at least one of the babies)**		
** Number of patients**	27,586	110,344
** Outcome events, ***n* **(%)**	4922 (17.84%)	18,593 (16.85%)
** Unadjusted OR (95% CI)**	1.07 (1.03–1.11)	
** Adjusted OR (95% CI) (Model 1)**	1.08 (1.05–1.12)	
** Adjusted OR (95% CI) (Model 2)**	1.06 (1.03–1.10)	
** Adjusted OR (95% CI) (Model 3)**	1.06 (1.03–1.10)	
** Adjusted OR (95% CI) (Model 4)**	1.00 (0.97–1.04)	
**Small for gestational age < 10th percentile (for at least one of the babies)**		
** Number of patients**	27,586	110,344
** Outcome events, ***n* **(%)**	1113 (4.03%)	4305 (3.90%)
** Unadjusted OR (95% CI)**	1.04 (0.97–1.11)	
** Adjusted OR (95% CI) (Model 1)**	1.01 (0.94–1.09)	
** Adjusted OR (95% CI) (Model 2)**	1.01 (0.94–1.09)	
** Adjusted OR (95% CI) (Model 3)**	1.00 (0.93–1.08)	
** Adjusted OR (95% CI) (Model 4)**	1.03 (0.96–1.11)	

^*^Record of PCOS/PCO/combination of symptom codes indicating a missed PCOS diagnosis based on Rotterdam criteria [(1) anovulation and (2) biochemical or symptomatic presentation of hyperandrogenism; Read code record of hair loss or hirsutism and a recorded measure of serum testosterone level ≥ 2.0 nmol/L was considered as symptomatic and biochemical presentation of hyperandrogenism, respectively]

*PCOS* Polycystic ovary syndrome, *CS* Caesarean section, *OR* Odds ratio

Model 1: Adjusted for age, ethnicity, and deprivation

Model 2: Adjusted for age, ethnicity, deprivation, baseline dysglycaemia, hypertension and thyroid disorders

Model 3: Adjusted for age, ethnicity, deprivation, baseline dysglycaemia, hypertension, thyroid disorders, and numbers of babies born at the delivery

Model 4: Adjusted for age, ethnicity, deprivation, baseline dysglycaemia, hypertension, thyroid disorders, numbers of babies born at the delivery, and pre-gravid body mass index

#### Mode of delivery

Compared to deliveries of women without PCOS, delivery of women with PCOS were more likely to occur by caesarean section [emergency: 12.59% vs 10.94%, elective/other/unspecified: 15.26% vs 13.85%] and less likely to occur by vaginal delivery [instrumental: 11.15% vs 11.39%, spontaneous/other/unspecified: 60.99% vs 63.82%)]. When serially adjusting for covariates, marginal attenuation in the effect estimate was observed, with the highest drop observed when adjusting for pre-gravid BMI. In the fully adjusted model, compared to spontaneous/other/unspecified vaginal delivery, delivery of women with PCOS was 4% at higher odds of being an instrumental vaginal delivery [aOR: 1.04 (1.00–1.09)], 7% at higher odds of being elective/other/unspecified caesarean section [aOR: 1.07 (1.03–1.12)] and 10% at higher odds of being emergency caesarean section [aOR: 1.10 (1.05–1.15)] compared to women without PCOS (Table 2). In the sensitivity analysis, among deliveries of women with a diagnostic code for PCOS and their matched delivery records, the increased odds for instrumental vaginal delivery was no longer evident and for elective/other/unspecified caesarean section was less pronounced [aOR: 1.00 (1.00–1.00) and 1.03 (1.02–1.03), respectively], while there was a more pronounced increased odds of emergency caesarean section delivery [aOR: 1.15 (1.02–1.30)] (Additional file 4).

#### Birthweight

The proportion of at least one of the babies in a single delivery being born with high birthweight (> 4000 g) did not differ significantly between delivery records of women with and without PCOS [9.82% vs 9.64%, OR: 1.02 (0.98–1.07), aOR: 0.97 (0.92–1.01)]. The proportion of low birthweight (< 2500 g) was significantly higher among deliveries of women with PCOS compared to women without PCOS (5.90% vs 5.35%), with an 11% increase in the crude odds of low birthweight [OR: 1.11 (1.05–1.18)]. However, this was insignificant in the fully adjusted model [aOR: 1.03 (0.95–1.13)] (Table 2).

In the sensitivity analysis, in the fully adjusted model, there was no increased risk of either high or low birthweight of babies born to mothers with PCOS compared to mothers without PCOS [aOR: 1.00 (0.88–1.13) and 1.03 (0.77–1.37), respectively] (Additional file 4).

When standardizing the birthweight using INTERGROWTH 21st project tools and considering the outcomes large and small for gestational age (LGA and SGA), there was a significant association between maternal PCOS and LGA babies in the unadjusted model [uOR: 1.07 (1.03–1.11)], which became non-significant when adjusting for pre-gravid BMI. There was no statistically significant association between maternal PCOS and odds of either LGA or SGA in the fully adjusted analysis [aOR: 1.00 (0.97–1.04) and 1.03 (0.96–1.11), respectively] (Table 3). In the fully adjusted sensitivity analysis, there was no significant association between maternal PCOS and LGA [aOR: 1.08 (0.99–1.18)], similar to the primary analysis; however, there was 26% lower odds of SGA in deliveries among women with PCOS compared to women without PCOS [aOR: 0.74 (0.59–0.94)] (Additional file 5).

#### Stillbirth

Among women with and without PCOS, the proportion of deliveries with stillbirth was 0.44% and 0.43%, respectively, and there was no significant difference in the crude or adjusted odds of stillbirth in either the primary or sensitivity analysis [aOR: 0.99 (0.81–1.21) and 0.52 (0.27–1.02), respectively].

## Discussion

### Main findings

In this retrospective cohort study of hospital-based delivery records, we found that women with PCOS were at an increased risk of preterm delivery and caesarean section compared to women without PCOS, even after accounting for several confounders including sociodemographic variables, pre-existing maternal conditions such as dysglycaemia, hypertension, and thyroid disorders, number of babies born at the delivery and pre-gravid BMI. Furthermore, we found that women with PCOS were crudely at an increased risk of delivering small babies weighing below 2.5 kg; however, the association disappeared after adjustment for gestational age. This was further supported by the absence of evidence of increased risk of babies born small for gestational age, suggesting that lower birthweight of babies born to mothers with PCOS was mediated by their lower gestational age at delivery. This also highlights the importance of standardising birthweight against gestational age using anthropometric reference data to define optimal foetal growth outcomes as opposed to using absolute birthweight. We also found that there was an increased risk of babies born large for gestational age among women with PCOS, but the association became insignificant with adjustment for pre-gravid BMI, suggesting that LGA is mediated by maternal pre-gravid BMI. There was no evidence of association between maternal PCOS and the risk of stillbirth.

### Strengths and limitations

Our study has many strengths including large sample size, and population-based data collected from routinely collected primary care records and hospital episode statistics birth records. One of the limitations might be the underdiagnosis of PCOS within the data source used. It is notable that across different settings, women with PCOS experience long delays in diagnosis and tend to report their symptoms multiple times prior to a diagnosis [5]. We therefore included women with a diagnostic code for PCO, or a combination of symptom codes indicating a missed PCOS diagnosis based on the Rotterdam criteria, which constituted 83% of the exposed women included in the primary analysis. This higher estimate of missed PCOS diagnosis in comparison to the literature [2, 4] may have introduced misclassification within the PCOS exposure group. Therefore, we performed a sensitivity analysis including only women with a diagnostic code for PCOS and their age-matched controls. Women with a diagnostic code for PCOS within primary care may reflect those with a severe phenotype associated with the combination of menstrual irregularity and androgen excess, who consulted their general practitioners for treatment and management [1]. In agreement with this, the results of our sensitivity analysis, restricted to women with a diagnostic code for PCOS and their matched controls, suggest a more profound and significant odds ratio for preterm, very preterm and extremely preterm delivery compared to results from our primary analysis.

A limitation of the study is the missing outcome data, for which we performed multiple imputation. Furthermore, information on some of the confounders including maternal education level, primigravidity were unavailable within the data source used. Another limitation of this study is the restriction of the eligible cohort to deliveries recorded within the hospital setting, thereby missing deliveries that happened elsewhere such as in non-NHS hospitals or in the home setting. This may affect the generalizability of our findings. However, 96% of deliveries in England are recorded within HES data [32].

Another limitation of the study is the absence of data on mode of conception; we were therefore unable to evaluate any effect modification attributable to in vitro fertilization when assessing the association between PCOS and risk of obstetric outcomes. The increased risk of obstetric outcomes among women with PCOS observed in our study may therefore be attributable to a combination of exposures to PCOS and in vitro fertilization, a prevalent mode of conception among women with PCOS.

We did not adjust for pregnancy-induced complications or gestational weight gain as these constitute intermediates between pre-pregnancy risk factors and obstetric outcomes. It is well established that women with PCOS are at an increased risk of developing antepartum complications such as gestational diabetes, pregnancy-induced hypertension and pre-eclampsia [33]. Considering the increased risk of preterm delivery conferred by these pregnancy complications [34, 35], it is possible that pregnancy complications formed the interlink between maternal PCOS and the risk of preterm delivery. Furthermore, caesarean section may be considered for the management of women presenting with suspected or established preterm labour [36]. This complex biological pathway mediated by pregnancy-induced complications could potentially explain the increased risk of preterm and operative delivery observed in our study.

### Comparison with existing literature

Our study is in agreement with existing reviews [37–39] and a recent Swedish nationwide cohort study [40] and confirms the association between maternal PCOS and preterm birth of varying degrees. However, the adjusted odds ratios observed in our study for preterm birth are modest compared to the odds ratios reported in the literature. This may be attributed to several factors including differences in the source population, exposure definition and residual confounding. Furthermore, genome-wide association studies have indicated a genetic polymorphism (EBF-1 gene) to be associated with both women’s likelihood of delivery preterm [41] and progression of PCOS [42], providing a plausible genetic explanation to our finding. In addition, a dysregulated hypothalamic–pituitary–adrenal (HPA) axis, as observed in both women with PCOS [43] and manifested during stress [44], has been associated with a modest increased risk of spontaneous preterm delivery, further supporting our findings.

Our study is also in agreement with reviews and cohort studies that suggest an increased risk of caesarean delivery [16, 37]. Our findings of absence of significant association of maternal PCOS with stillbirth is supported by Roos et.al. [16], while a more recent study by Valgeirsdottir et.al. [45], suggests a 50% increased risk of stillbirth among women with PCOS, although the exposure ascertainment within the study suffers from misclassification due to inclusion of women with anovulation as well as women with PCOS.

### Implications

With a PCOS diagnosis, women have expressed concerns about infertility and pregnancy [46], and would benefit from the awareness of their pregnancy and delivery-related risks, and evidence-based surveillance and care to avert these risks. Future research is needed to understand the pathophysiological underpinnings of maternal PCOS on the risk of obstetric outcomes, so that interventions can be designed to reduce these risks.

### Conclusion

Women with PCOS are at an increased risk of obstetric outcomes including preterm and operative delivery. Association with low birthweight maybe mediated by lower gestational age at delivery.

## Supplementary Information

**Additional file 1.** Read codes for exposure ascertainment

**Additional file 2.** Read codes for outcome ascertainment

**Additional file 3.** Baseline characteristics of women with PCOS and age matched controls – Sensitivity Analysis

**Additional file 4.** Risk of primary obstetric outcomes among women with PCOS compared to women without PCOS – Sensitivity Analysis

**Additional file 5.** Risk of secondary obstetric outcomes among women with PCOS compared to women without PCOS – Sensitivity Analysis

## Data Availability

The data that support the findings of this study are available from CPRD, but restrictions apply to the availability of this data, which was used under license for the current study, and so is not publicly available.

## Abbreviations

PCOS: Polycystic ovary syndrome
CPRD: Clinical Practice Research Datalink
HES: Hospital Episode Statistics
NHS: National Health Service
PCO: Polycystic ovary
OPCS: Operating Procedure Codes Supplement
BMI: Body mass index
SD: Standard deviation
IQR: Interquartile range
OR: Odds ratio
RECORD: REporting of studies Conducted using Observational Routinely-collected health Data
IMD: Index of multiple deprivation
LGA: Large for gestational age
SGA: Small for gestational age
HPA: Hypothalamic-pituitary-adrenal
