# Supplementary material for: Polycystic ovary syndrome and risk of adverse obstetric outcomes: a retrospective population-based matched cohort study in England
Source: BMC Med. 2022 Aug 30;20:298. doi: 10.1186/s12916-022-02473-3 (PMC9425992; doi:10.1186/s12916-022-02473-3)
Supplement: Supplementary file 1 — Additional file 1. Read codes for exposure ascertainment [file 12916_2022_2473_MOESM1_ESM.docx]

**Supplementary Table 1A: Read codes for exposure ascertainment – PCOS**

| **READ CODE** | **DESCRIPTION** |
| --- | --- |
| C165.00 | Polycystic ovarian syndrome |
| C164.12 | Stein - Leventhal syndrome |

**Supplementary Table 1B: Read codes for exposure ascertainment – PCO**

| **READ CODE** | **DESCRIPTION** |
| --- | --- |
| C164.00 | Polycystic ovaries |
| C164.13 | Multicystic ovaries |

**Supplementary Table 1C: Read codes for exposure ascertainment – anovulation**

| **READ CODE** | **DESCRIPTION** |
| --- | --- |
| K590.11 | Amenorrhoea |
| K591100 | Oligomenorrhoea |
| K590100 | Secondary amenorrhoea |
| K5B0.00 | Female infertility of anovulatory origin |
| K590.00 | Absence of menstruation |
| K591300 | Secondary oligomenorrhoea |
| K591000 | Hypomenorrhoea |
| K5B0.11 | Anovular cycle |
| 1571.00 | H/O: amenorrhoea |
| K591.00 | Scanty or infrequent menstruation |
| K591.11 | Infrequent menstruation |
| K591z00 | Scanty or infrequent menstruation NOS |
| K590z00 | Amenorrhoea NOS |
| K5B0100 | Secondary anovulatory infertility |
| K5B0z00 | Female infertility of anovulatory origin NOS |

**Supplementary Table 1D: Read codes for exposure ascertainment – hair loss**

| **READ CODE** | **DESCRIPTION** |
| --- | --- |
| M240000 | Alopecia unspecified |
| M240.00 | Alopecia |
| M240012 | Hair loss |
| 22D7.11 | O/E - alopecia |
| M240z00 | Alopecia NOS |
| M240200 | Male pattern alopecia |
| 1N02.00 | C/O: hair loss |
| M240300 | Frontal alopecia of women |
| 22D4.00 | O/E - loss of hair |
| M240H00 | Alopecia seborrhoeica |
| Myu6300 | [X]Other androgenic alopecia |
| M240D00 | Marginal alopecia |

**Supplementary Table 1E: Read codes for exposure ascertainment – hirsutism**

| **READ CODE** | **DESCRIPTION** |
| --- | --- |
| M241.00 | Hirsutism - hypertrichosis |
| 22D8000 | O/E - facial hair |
| 22D8.00 | O/E - hirsutism |
