# Supplementary material for: Polycystic ovary syndrome and risk of adverse obstetric outcomes: a retrospective population-based matched cohort study in England
Source: BMC Med. 2022 Aug 30;20:298. doi: 10.1186/s12916-022-02473-3 (PMC9425992; doi:10.1186/s12916-022-02473-3)
Supplement: Supplementary file 2 — Additional file 2. Read codes for outcome ascertainment [file 12916_2022_2473_MOESM2_ESM.docx]

**Supplementary Table 2A: ICD-10 codes for outcome ascertainment – preterm birth**

| **ICD-10 CODE** | **DESCRIPTION** |
| --- | --- |
| O60 | Preterm labour and delivery |
| O60.1 | Preterm spontaneous labour with preterm delivery |
| O60.2 | Preterm spontaneous labour with term delivery |
| O60.3 | Preterm delivery without spontaneous labour |
| P07.2 | Extreme immaturity |
| P07.3 | Other preterm infants |
| P59.0 | Neonatal jaundice associated with preterm delivery |
| P61.2 | Anaemia of prematurity |

**Supplementary Table 2B: OPCS codes for outcome ascertainment – mode of delivery**

|  | **OPCS CODE** | **DESCRIPTION** |
| --- | --- | --- |
| Emergency Caesarean section | R180 | Other caesarean delivery |
|  | R181 | Upper uterine segment caesarean delivery NEC |
|  | R182 | Lower uterine segment caesarean delivery NEC |
|  | R188 | Other specified other caesarean delivery |
|  | R189 | Unspecified other caesarean delivery |
| Elective or other unspecified Caesarean section | R170 | Elective caesarean delivery |
|  | R171 | Elective upper uterine segment caesarean delivery |
|  | R172 | Elective lower uterine segment caesarean delivery |
|  | R178 | Other specified elective caesarean delivery |
|  | R179 | Unspecified elective caesarean delivery |
|  | R251 | Caesarean hysterectomy |
| Instrumental vaginal delivery | R210 | Forceps cephalic delivery |
|  | R211 | High forceps cephalic delivery with rotation |
|  | R212 | High forceps cephalic delivery NEC |
|  | R213 | Mid forceps cephalic delivery with rotation |
|  | R214 | Mid forceps cephalic delivery NEC |
|  | R215 | Low forceps cephalic delivery |
|  | R218 | Other specified forceps cephalic delivery |
|  | R219 | Unspecified forceps cephalic delivery |
|  | R220 | Vacuum delivery |
|  | R221 | High vacuum delivery |
|  | R222 | Low vacuum delivery |
|  | R223 | Vacuum delivery before full dilation of cervix |
|  | R228 | Other specified vacuum delivery |
|  | R229 | Unspecified vacuum delivery |
| Spontaneous or other unspecified vaginal delivery | R201 | Spontaneous breech delivery |
|  | R230 | Cephalic vaginal delivery with abnormal presentation of head at delivery without instrument |
|  | R231 | Manipulative cephalic vaginal delivery with abnormal presentation of head at delivery without instrument |
|  | R232 | Non-manipulative cephalic vaginal delivery with abnormal presentation of head at delivery without instrument |
|  | R238 | Other specified cephalic vaginal delivery with abnormal presentation of head at delivery without instrument |
|  | R239 | Unspecified cephalic vaginal delivery with abnormal presentation of head at delivery without instrument |
|  | R240 | Normal delivery |
|  | R249 | All normal delivery |
|  | R190 | Breech extraction delivery |
|  | R191 | Breech extraction delivery with version |
|  | R198 | Other specified breech extraction delivery |
|  | R199 | Unspecified breech extraction delivery |
|  | R200 | Other breech delivery |
|  | R208 | Other specified other breech delivery |
|  | R209 | Unspecified other breech delivery |
|  | R202 | Assisted breech delivery |
|  | R252 | Destructive operation to facilitate delivery |
|  | R258 | Other specified other methods of delivery |
|  | R259 | Unspecified other methods of delivery |

**Supplementary Table 2C: ICD-10 codes for outcome ascertainment – mode of delivery**

|  | **ICD-10 CODE** | **DESCRIPTION** |
| --- | --- | --- |
| Emergency Caesarean section | O82.1 | Delivery by emergency caesarean section |
| Elective or other unspecified Caesarean section | O82.0 | Delivery by elective caesarean section |
|  | O82.2 | Delivery by caesarean hysterectomy |
|  | O82.8 | Other single delivery by caesarean section |
|  | O82.9 | Delivery by caesarean section unspecified |
|  | O84.2 | Multiple delivery all by caesarean section |
|  | O83.3 | Delivery of viable fetus in abdominal pregnancy |
| Instrumental vaginal delivery | O81.0 | Low forceps delivery |
|  | O81.1 | Mid-cavity forceps delivery |
|  | O81.2 | Mid-cavity forceps with rotation |
|  | O81.3 | Other and unspecified forceps delivery |
|  | O81.4 | Vacuum extractor delivery |
|  | O81.5 | Delivery by combination of forceps and vacuum extractor |
|  | O84.1 | Multiple delivery all by forceps and vacuum extractor |
| Spontaneous or other unspecified vaginal delivery | O80.0 | Spontaneous vertex delivery |
|  | O80.1 | Spontaneous breech delivery |
|  | O80.8 | Other single spontaneous delivery |
|  | O80.9 | Single spontaneous delivery unspecified |
|  | O84.0 | Multiple delivery all spontaneous |
|  | O83.0 | Breech extraction |
|  | O83.1 | Other assisted breech delivery |
|  | O83.2 | Other manipulation-assisted delivery |
|  | O83.8 | Other specified assisted single delivery |
|  | O83.9 | Assisted single delivery unspecified |
|  | O84.8 | Other multiple delivery |
|  | O84.9 | Multiple delivery unspecified |

**Supplementary Table 2D: ICD-10 codes for outcome ascertainment – stillbirth**

| **ICD-10_CODE** | **DESCRIPTION** |
| --- | --- |
| Z37.1 | Single stillbirth |

**Supplementary Table 2E: ICD-10 codes for outcome ascertainment – High birthweight**

| **ICD-10_CODE** | **DESCRIPTION** |
| --- | --- |
| P08.0 | Exceptionally large baby |
