# Supplementary material for: Polycystic ovary syndrome and risk of adverse obstetric outcomes: a retrospective population-based matched cohort study in England
Source: BMC Med. 2022 Aug 30;20:298. doi: 10.1186/s12916-022-02473-3 (PMC9425992; doi:10.1186/s12916-022-02473-3)
Supplement: Supplementary file 3 — Additional file 3. Baseline characteristics of women with PCOS and age matched controls – Sensitivity Analysis [file 12916_2022_2473_MOESM3_ESM.docx]

**Supplementary Table 3: Baseline characteristics of women with PCOS and age matched controls – Sensitivity Analysis**

| **Variables** | **Deliveries of women  with PCOS*** | **Age matched deliveries of women  without PCOS** |
| --- | --- | --- |
| **All** | **(n=4,559)** | **(n=18,236)** |
| **Age at delivery [Mean (SD)]** | 31.13 (5.10) | 31.12 (5.05) |
| **Age at delivery [Median (IQR)]** | 31.00 (27.00-34.00) | 31.00 (27.00-34.00) |
| **Age categories, n (%)** |  |  |
| **14 - 20 years** | 34 (0.75) | 137 (0.75) |
| **20 - 30 years** | 1850 (40.58) | 7302 (40.04) |
| **30 - 40 years** | 2491 (54.64) | 10107 (55.42) |
| **40 - 50 years** | 184 (4.04) | 690 (3.78) |
| **Pre-gravid BMI [Mean (SD)]** | 28.34 (6.94) | 25.12 (5.46) |
| **Pre-gravid BMI [Median (IQR)]** | 27.00 (22.00-32.00) | 23.00 (21.00-27.00) |
| **BMI Categories, n (%)** |  |  |
| **<25 kg/m2** | 1656 (36.32) | 9863 (54.09) |
| **25-30 kg/m2** | 1115 (24.46) | 4258 (23.35) |
| **30-35 kg/m2** | 786 (17.24) | 1680 (9.21) |
| **35-40 kg/m2** | 477 (10.46) | 641 (3.52) |
| **>40 kg/m2** | 275 (6.03) | 358 (1.96) |
| **Missing** | 250 (5.48) | 1436 (7.87) |
| **IMD, n (%)** |  |  |
| **1 Most deprived)** | 552 (12.11) | 2218 (12.16) |
| **2** | 457 (10.02) | 1783 (9.78) |
| **3** | 460 (10.09) | 1866 (10.23) |
| **4** | 445 (9.76) | 1774 (9.73) |
| **5** | 464 (10.18) | 1970 (10.80) |
| **6** | 377 (8.27) | 1750 (9.60) |
| **7** | 449 (9.85) | 1720 (9.43) |
| **8** | 450 (9.87) | 1724 (9.45) |
| **9** | 458 (10.05) | 1668 (9.15) |
| **10 (Least deprived)** | 445 (9.76) | 1753 (9.61) |
| **Missing** | 2 (0.04) | 10 (0.05) |
| **Ethnicity, n (%)** |  |  |
| **White Caucasian** | 2401 (52.67) | 8445 (46.31) |
| **South Asian** | 370 (8.12) | 573 (3.14) |
| **Black Afro-Caribbean** | 286 (6.27) | 854 (4.68) |
| **Mixed Race** | 46 (1.01) | 97 (0.53) |
| **Others** | 118 (2.59) | 416 (2.28) |
| **Missing** | 1338 (29.35) | 7851 (43.05) |
| **Record of symptoms and measurements at baseline, n (%)** |  |  |
| **PCO** | 1580 (34.66) | 0 (0) |
| **Hair Loss** | 295 (6.47) | 425 (2.33) |
| **Hirsutism** | 491 (10.77) | 120 (0.66) |
| **Anovulation** | 1910 (41.90) | 1864 (10.22) |
| **High Testosterone (serum testosterone level ≥ 2.0 nmol/L)** | 835 (18.32) | 92 (0.50) |
| **Other comorbidities, n (%)** |  |  |
| **Type 2 diabetes** | 142 (3.11) | 207 (1.14) |
| **Prediabetes** | 265 (5.81) | 333 (1.83) |
| **Hypertension** | 114 (2.50) | 212 (1.16) |
| **Thyroid disorders** | 251 (5.51) | 415 (2.28) |
| **Pregnancy related variables** | 265 (5.81) | 333 (1.83) |
| **Number of babies at the delivery, n (%)** |  |  |
| **1** | 4492 (98.53) | 17908 (98.20) |
| **2** | 64 (1.40) | 318 (1.74) |
| **3** | 3 (0.07) | 9 (0.05) |
| **4+** | 0 (0) | 1 (0.01) |

*Patients with a diagnostic code for PCOS only

PCOS: Polycystic Ovary Syndrome; PCO: Polycystic ovaries; SD: Standard Deviation; IQR: Interquartile Range; BMI: Body Mass Index; IMD: Index of Multiple Deprivation
