# Supplementary material for: Polycystic ovary syndrome and risk of adverse obstetric outcomes: a retrospective population-based matched cohort study in England
Source: BMC Med. 2022 Aug 30;20:298. doi: 10.1186/s12916-022-02473-3 (PMC9425992; doi:10.1186/s12916-022-02473-3)
Supplement: Supplementary file 4 — Additional file 4. Risk of primary obstetric outcomes among women with PCOS compared to women without PCOS – Sensitivity Analysis [file 12916_2022_2473_MOESM4_ESM.docx]

**Supplementary Table 4: Risk of primary obstetric outcomes among women with PCOS compared to women without PCOS – Sensitivity Analysis**

| **Outcomes** | **Deliveries of women  with PCOS*** | **Age matched deliveries of women  without PCOS** |
| --- | --- | --- |
| **Preterm** |  |  |
| **Number of patients** | 4559 | 18236 |
| **Outcome events, n (%)** | 391 (8.58%) | 1223 (6.71%) |
| **Unadjusted OR (95% CI)** | 1.31 (1.15-1.48) | |
| **Adjusted OR (95% CI) (Model 1)** | 1.29 (1.11-1.44) | |
| **Adjusted OR (95% CI) (Model 2)** | 1.23 (1.06-1.44) | |
| **Adjusted OR (95% CI) (Model 3)** | 1.29 (1.13-1.47) | |
| **Adjusted OR (95% CI) (Model 4)** | 1.31 (1.13-1.52) | |
| **Model of delivery** |  |  |
| **Number of patients** | 4559 | 18236 |
| **Outcome events, n (%)** |  |  |
| **Emergency CS** | 610 (13.38%) | 1986 (10.89%) |
| **Elective/Other/Unspecified CS** | 798 (17.50%) | 2535 (13.90%) |
| **Instrumental Vaginal** | 536 (11.76%) | 2051 (11.25%) |
| **Spontaneous/Other/Unspecified Vaginal** | 2615 (57.36%) | 11664 (63.96%) |
| **Unadjusted OR (95% CI)** |  |  |
| **Emergency CS** | 1.37 (1.23-1.53) | |
| **Elective/Other/Unspecified CS** | 1.40 (1.27-1.55) | |
| **Instrumental Vaginal** | 1.17 (1.05-1.30) | |
| **Spontaneous/Other/Unspecified Vaginal** | Ref | |
| **Adjusted OR (95% CI) (Model 1)** |  |  |
| **Emergency CS** | 1.37 (1.23-1.54) | |
| **Elective/Other/Unspecified CS** | 1.38 (1.25-1.52) | |
| **Instrumental Vaginal** | 1.17 (1.05-1.30) | |
| **Spontaneous/Other/Unspecified Vaginal** | Ref | |
| **Adjusted OR (95% CI) (Model 2)** |  |  |
| **Emergency CS** | 1.31 (1.17-1.47) | |
| **Elective/Other/Unspecified CS** | 1.36 (1.23-1.50) | |
| **Instrumental Vaginal** | 1.18 (1.06-1.32) | |
| **Spontaneous/Other/Unspecified Vaginal** | Ref | |
| **Adjusted OR (95% CI) (Model 3)** |  |  |
| **Emergency CS** | 1.32 (1.17-1.48) | |
| **Elective/Other/Unspecified CS** | 1.37 (1.24-1.51) | |
| **Instrumental Vaginal** | 1.18 (1.06-1.32) | |
| **Spontaneous/Other/Unspecified Vaginal** | Ref | |
| **Adjusted OR (95% CI) (Model 4)** |  |  |
| **Emergency CS** | 1.17 (1.04-1.31) | |
| **Elective/Other/Unspecified CS** | 1.25 (1.13-1.38) | |
| **Instrumental Vaginal** | 1.24 (1.11-1.38) | |
| **Spontaneous/Other/Unspecified Vaginal** | Ref | |
| **Adjusted OR (95% CI) (Model 5)** |  |  |
| **Emergency CS** | 1.15 (1.02-1.30) | |
| **Elective/Other/Unspecified CS** | 1.03 (1.02-1.03) | |
| **Instrumental Vaginal** | 1.00 (1.00-1.00) | |
| **Spontaneous/Other/Unspecified Vaginal** | Ref | |
| **High birthweight >4 kg (for at least one of the baby)** |  |  |
| **Number of patients** | 4559 | 18236 |
| **Outcome events, n (%)** | 500 (10.97%) | 1846 (10.12%) |
| **Unadjusted OR (95% CI)** | 1.09 (0.98-1.22) | |
| **Adjusted OR (95% CI) (Model 1)** | 1.12 (0.95-1.33) | |
| **Adjusted OR (95% CI) (Model 2)** | 1.11 (0.94-1.31) | |
| **Adjusted OR (95% CI) (Model 3)** | 1.11 (0.94-1.31) | |
| **Adjusted OR (95% CI) (Model 4)** | 0.97 (0.84-1.12) | |
| **Adjusted OR (95% CI) (Model 5)** | 1.00 (0.88-1.13) | |
| **Low birthweight <2.5 kg (for at least one of the baby)** |  |  |
| **Number of patients** | 4559 | 18236 |
| **Outcome events, n (%)** | 277 (6.08%) | 1001 (5.49%) |
| **Unadjusted OR (95% CI)** | 1.11 (0.96-1.29) | |
| **Adjusted OR (95% CI) (Model 1)** | 1.09 (0.95-1.24) | |
| **Adjusted OR (95% CI) (Model 2)** | 1.06 (0.93-1.21) | |
| **Adjusted OR (95% CI) (Model 3)** | 1.09 (0.94-1.26) | |
| **Adjusted OR (95% CI) (Model 4)** | 1.18 (1.00-1.39) | |
| **Adjusted OR (95% CI) (Model 5)** | 1.03 (0.77-1.37) | |
| **Stillbirth** |  |  |
| **Number of patients** | 4559 | 18236 |
| **Outcome events, n (%)** | 16 (0.35%) | 82 (0.45%) |
| **Unadjusted OR (95% CI)** | 0.78 (0.46-1.32) | |
| **Adjusted OR (95% CI) (Model 1)** | 0.74 (0.44-1.26) | |
| **Adjusted OR (95% CI) (Model 2)** | 0.70 (0.27-1.77) | |
| **Adjusted OR (95% CI) (Model 3)** | 0.66 (0.25-1.72) | |
| **Adjusted OR (95% CI) (Model 4)** | 0.52 (0.27-1.02) | |

*Patients with a diagnostic code for PCOS only

PCOS: Polycystic Ovary Syndrome; CS: Caesarean Section; OR: Odds Ratio

Model 1: Adjusted for age, ethnicity, and deprivation

Model 2: Adjusted for age, ethnicity, deprivation, baseline dysglycaemia, hypertension and thyroid disorders

Model 3: Adjusted for age, ethnicity, deprivation, baseline dysglycaemia, hypertension, thyroid disorders, and numbers of babies born at the delivery

Model 4: Adjusted for age, ethnicity, deprivation, baseline dysglycaemia, hypertension, thyroid disorders, numbers of babies born at the delivery, and pre-gravid body mass index

Model 5: Adjusted for age, ethnicity, deprivation, baseline dysglycaemia, hypertension, thyroid disorders, numbers of babies born at the delivery, pre-gravid body mass index, and gestational age
