# Supplementary material for: Polycystic ovary syndrome and risk of adverse obstetric outcomes: a retrospective population-based matched cohort study in England
Source: BMC Med. 2022 Aug 30;20:298. doi: 10.1186/s12916-022-02473-3 (PMC9425992; doi:10.1186/s12916-022-02473-3)
Supplement: Supplementary file 5 — Additional file 5. Risk of secondary obstetric outcomes among women with PCOS compared to women without PCOS – Sensitivity Analysis [file 12916_2022_2473_MOESM5_ESM.docx]

**Supplementary Table 5: Risk of secondary obstetric outcomes among women with PCOS compared to women without PCOS – Sensitivity Analysis**

| **Outcomes** | **Deliveries of women  with PCOS*** | **Age matched deliveries of women  without PCOS** |
| --- | --- | --- |
| **Very Preterm (<32 weeks of gestational age at delivery)** |  |  |
| **Number of patients** | 4559 | 18236 |
| **Outcome events, n (%)** | 125 (2.74%) | 346 (1.90%) |
| **Unadjusted OR (95% CI)** | 1.46 (1.16-1.84) | |
| **Adjusted OR (95% CI) (Model 1)** | 1.44 (0.98-2.11) | |
| **Adjusted OR (95% CI) (Model 2)** | 1.44 (0.87-2.39) | |
| **Adjusted OR (95% CI) (Model 3)** | 1.43 (0.98-2.08) | |
| **Adjusted OR (95% CI) (Model 4)** | 1.42 (0.88-2.31) | |
| **Extremely preterm (<28 weeks of gestational age at delivery)** |  |  |
| **Number of patients** | 4559 | 18236 |
| **Outcome events, n (%)** | 59 (1.29%) | 137 (0.75%) |
| **Unadjusted OR (95% CI)** | 1.73 (1.25-2.41) | |
| **Adjusted OR (95% CI) (Model 1)** | 1.86 (1.33-2.60) | |
| **Adjusted OR (95% CI) (Model 2)** | 1.97 (1.43-2.71) | |
| **Adjusted OR (95% CI) (Model 3)** | 1.93 (1.23-3.05) | |
| **Adjusted OR (95% CI) (Model 4)** | 1.86 (1.31-2.65) | |
| **Large for gestational age >90th percentile (for at least one of the babies)** |  |  |
| **Number of patients** | 4559 | 18236 |
| **Outcome events, n (%)** | 925 (20.29%) | 3161 (17.33%) |
| **Unadjusted OR (95% CI)** | 1.22 (1.11-1.33) | |
| **Adjusted OR (95% CI) (Model 1)** | 1.23 (1.13-1.34) | |
| **Adjusted OR (95% CI) (Model 2)** | 1.21 (1.11-1.32) | |
| **Adjusted OR (95% CI) (Model 3)** | 1.21 (1.11-1.31) | |
| **Adjusted OR (95% CI) (Model 4)** | 1.08 (0.99-1.18) | |
| **Small for gestational age <10th percentile (for at least one of the babies)** |  |  |
| **Number of patients** | 4559 | 18236 |
| **Outcome events, n (%)** | 146 (3.20%) | 753 (4.13%) |
| **Unadjusted OR (95% CI)** | 0.77 (0.64-0.92) | |
| **Adjusted OR (95% CI) (Model 1)** | 0.72 (0.57-0.91) | |
| **Adjusted OR (95% CI) (Model 2)** | 0.72 (0.55-0.95) | |
| **Adjusted OR (95% CI) (Model 3)** | 0.70 (0.53-0.93) | |
| **Adjusted OR (95% CI) (Model 4)** | 0.74 (0.59-0.94) | |

*Patients with a diagnostic code for PCOS only

PCOS: Polycystic Ovary Syndrome; CS: Caesarean Section; OR: Odds Ratio

Model 1: Adjusted for age, ethnicity, and deprivation

Model 2: Adjusted for age, ethnicity, deprivation, baseline dysglycaemia, hypertension and thyroid disorders

Model 3: Adjusted for age, ethnicity, deprivation, baseline dysglycaemia, hypertension, thyroid disorders, and numbers of babies born at the delivery

Model 4: Adjusted for age, ethnicity, deprivation, baseline dysglycaemia, hypertension, thyroid disorders, numbers of babies born at the delivery, and pre-gravid body mass index
